# MR elastography following focused ultrasound treatment of uterine fibroids

**DOI:** 10.1186/2050-5736-2-S1-A13

**Published:** 2014-12-10

**Authors:** Gina Hesley, Krzysztof Gorny, David Woodrum

**Affiliations:** 1 Mayo Clinic, Rochester, MN, USA

## Background

Magnetic Resonance Elastography (MRE) is a technique in which shear waves are induced in soft tissues and are imaged by Magnetic Resonance (MR) is to assess tissue stiffness. While gadolinium has served as the standard for assessing immediate post treatment changes in the fibroids, some patients may have allergies to gadolinium preventing use of this contrast agent. Additionally, some patients may experience transient reduction in vascularity to non-treated fibroid tissue due to vessel spasm or other factors, which could possibly lead to overestimation of the treated volume. As of now, no studies have been performed to assess the mechanical changes associated with thermal damage to fibroid tissues induced by the MR-guided focused ultrasound (MRgFUS) treatment. The purpose of this feasibility study was to evaluate whether shear waves can be propagated through the uterus and distinguish fibroids from normal uterine tissue and to evaluate for any post changes in follow up MR exams.

## Materials and methods

A feasibility study was conducted by adding a MRE protocol to routine pre-treatment MRI with gadolinium for patients undergoing MRgFUS. The MRE was repeated after the MRgFUS and tissue stiffness in the treated volume, pre- and 2 year post-MRgFUS was recorded.

## Results

Feasibility testing has shown the ability to propagate shear waves through the uterus. Initial data from the 3 available studies indicate fibroids after treatment demonstrate an increase in stiffness from baseline (for example from 3.995 kPA to 7.725 kPA) and that post treatment fibroids are more easily distinguished from normal uterine tissue than on pre-treatment MRE imaging.

## Conclusion

Early results indicate promise for the use of MRE as an assessment of post MRgFUS changes in treated fibroids. However, continued optimization in technique and more data are needed to validate these early findings.

Investigator time was funded by NIH HD RC1063312 and R01060503

